# An integrated genomic and transcriptomic survey of mucormycosis-causing fungi

**DOI:** 10.1038/ncomms12218

**Published:** 2016-07-22

**Authors:** Marcus C. Chibucos, Sameh Soliman, Teclegiorgis Gebremariam, Hongkyu Lee, Sean Daugherty, Joshua Orvis, Amol C. Shetty, Jonathan Crabtree, Tracy H. Hazen, Kizee A. Etienne, Priti Kumari, Timothy D. O'Connor, David A. Rasko, Scott G. Filler, Claire M. Fraser, Shawn R. Lockhart, Christopher D. Skory, Ashraf S. Ibrahim, Vincent M. Bruno

**Affiliations:** 1 Department of Microbiology and Immunology, University of Maryland School of Medicine, Baltimore, Maryland 21201, USA; 2 Institute for Genome Sciences, University of Maryland School of Medicine, Baltimore, Maryland 21201, USA; 3 Division of Infectious Diseases, Los Angeles Biomedical Research Institute, Harbor-UCLA Medical Center, Torrance, California 90502, USA; 4 Fungal Reference Laboratory, Mycotic Diseases Branch, Centers for Disease Control and Prevention, Atlanta, Georgia 30333, USA; 5 Department of Medicine, University of Maryland School of Medicine, Baltimore, Maryland 21201, USA; 6 David Geffen School of Medicine at UCLA, Los Angeles, California 90502, USA; 7 National Center for Agriculture Utilization Research, USDA, Agricultural Research Service, Peoria, Illinois 61604, USA

## Abstract

Mucormycosis is a life-threatening infection caused by Mucorales fungi. Here we sequence 30 fungal genomes, and perform transcriptomics with three representative *Rhizopus* and *Mucor* strains and with human airway epithelial cells during fungal invasion, to reveal key host and fungal determinants contributing to pathogenesis. Analysis of the host transcriptional response to Mucorales reveals platelet-derived growth factor receptor B (PDGFRB) signaling as part of a core response to divergent pathogenic fungi; inhibition of PDGFRB reduces Mucorales-induced damage to host cells. The unique presence of CotH invasins in all invasive Mucorales, and the correlation between CotH gene copy number and clinical prevalence, are consistent with an important role for these proteins in mucormycosis pathogenesis. Our work provides insight into the evolution of this medically and economically important group of fungi, and identifies several molecular pathways that might be exploited as potential therapeutic targets.

## Abstract (web summary)

Fungi of the order Mucorales can cause life-threatening infections. Here, Chibucos *et al*. present genomic and transcriptomic analyses of a diverse set of Mucorales fungi, shedding light on their evolution and identifying potential therapeutic targets in the pathogens and the host.

## Graphical abstract

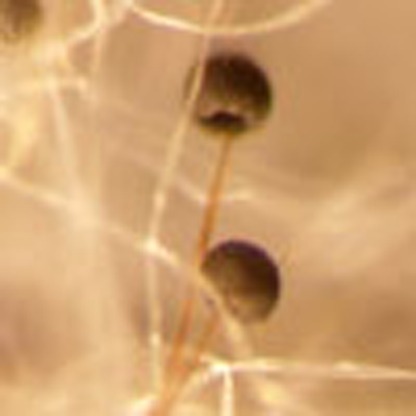

Mucormycoses are deadly human infections caused by fungi belonging to the subphylum Mucoromycotina, order Mucorales^1^,^2^. *Rhizopus* spp. are the most common organisms isolated from patients with mucormycosis and are responsible for ∼70% of all cases of this disease^1^,^2^,^3^. *Mucor* spp. and *Lichtheimia* spp. are also a significant cause of fungal infections in Europe with each causing ∼20% of the cases^4^, while *Apophysomyces* spp. are common clinical isolates in India^5^. Fungal species currently belonging to both the Mucormycotina and Entomophthoromycotina subphyla were formerly considered to be members of the zygomycota taxon based on morphological similarities, sexual reproductive structures, life cycle and ecology. However, following reanalysis of their evolutionary relationship using molecular phylogenetic techniques, these two taxa have been separated^6^. While mucormycoses are often invasive infections, entomophthoramycoses, caused by members of the subphylum Entomophthoromycotina, order Entomophthorales, are only known to be superficial infections^7^.

The major risk factors for mucormycosis include uncontrolled diabetes mellitus in ketoacidosis, other forms of metabolic acidosis, treatment with corticosteroids, solid organ or bone marrow transplantation, neutropenia, trauma and burns (for example, wounded soldiers in Iraq and Afghanistan), malignant haematological disorders and deferoxamine therapy in patients receiving haemodialysis^2^,^8^,^9^. Cutaneous necrotizing soft tissue mucormycosis outbreaks in otherwise healthy individuals have also been known to follow natural disasters, as evidenced by the *Apophysomyces* infections, usually associated with trauma, following the tsunami that devastated Indonesia in 2004 (ref. 10) and the tornadoes that occurred in Joplin, Missouri, USA in June 2011 (ref. 11).

The standard therapy for mucormycosis includes reversal of the underlying predisposing factors (if possible), wide-spread surgical debridement of the infected area^8^ and antifungal therapy^2^,^9^,^12^. In the absence of surgical removal of the infected focus (such as excision of the eye in patients with rhinocerebral mucormycosis), antifungal therapy alone is rarely curative^9^,^12^. Even when surgical debridement is combined with high-dose amphotericin B, the mortality associated with mucormycosis is >50% (ref. 12). In patients with prolonged neutropenia, disseminated disease or central nervous system infection mortality is 90–100% (refs 13, 14, 15).

The unacceptably high mortality rate, limited options for therapy and the extreme morbidity of highly disfiguring surgical therapy make it imperative to look for alternative strategies to treat and prevent these infections. While the genomes of some Mucorales isolates have been sequenced, there remains a paucity of genome data and comparative analyses for this group of fungal pathogens^11^,^16^,^17^,^18^,^19^,^20^,^21^,^22^,^23^,^24^,^25^. In this work, we utilize an integrated genomics approach to understand the population genetics, evolution and phylogeny of this understudied group of fungi, and identify potential therapeutic targets in the pathogen and the host.

## Results

### Genome sequence and annotation of Mucorales

To identify both common and taxa-specific genetic elements that contribute to pathogenesis, we sequenced, assembled and annotated the genomes of 27 isolates from the order Mucorales, including representative isolates of 15 different species from nine different genera: *Apophysomyces*, *Cokeromyces*, *Cunninghamella*, *Mucor*, *Rhizomucor*, *Rhizopus*, *Saksenaea*, *Syncephalastrum* and *Umbelopsis* (Table 1; Supplementary Data 1). We performed the same on the genomes of three strains from the order Entomophthorales: *Basidiobolus heterosporus*, *Basidiobolus. meristosporus* and *Conidiobolus incongruus* (*C. incongruus*). Since, these species are not known to cause invasive infections in humans, a comparative analysis of these strains with Mucorales strains will help to understand the genetic requirements for invasive disease. The majority of the strains sequenced in this study are clinical isolates with the exception of the *Apophysomyces elegans*, *Rhizopus stolonifer* and *B. heterosporus* strains. The specific source of each strain is listed in (Supplementary Data 1). All strains were sequenced using the Illumina HiSeq2000 platform and each assembly was created *de novo*.

We performed structural and functional annotation of each newly sequenced genome and used these annotations to perform downstream comparative genomic analyses (Supplementary Data 2). A total of 41 genomes were included in our comparative analysis: the 30 presented here plus 11 previously published genomes: two *Lichtheimia corymbifera*^16^, two *Lichtheimia ramosa*^16^, one *Mortierella alpina* (*M. alpina*)^26^, one *Rhizopus chinensis*^25^, one *Mucor velutinosus*^22^, one *Mucor circinelloides* (*M. circinelloides*)^22^, one *Mucor racemosus* (*M. racemosus*)^22^, one *Mucor indicus* (*M. indicus*)^22^ and the original *Rhizopus delemar* 99-880 (ref. 20). Among these 11 additional strains, 8 are clinical isolates while 3 (*L. corymbifera* CDC-B2541, *M. racemosus* CDC-B9645 and *Rhizopus microsporus* var. *chinensis*) are environmental isolates. To prevent our analyses from being limited by varying approaches to genome annotation, we used our annotation pipeline to annotate (or re-annotate in the case of *R. delemar* 99-880) the 11 previously published genomes (Supplementary Data 2).

### Phylogenetic analysis of Mucorales

**Figure 1:**
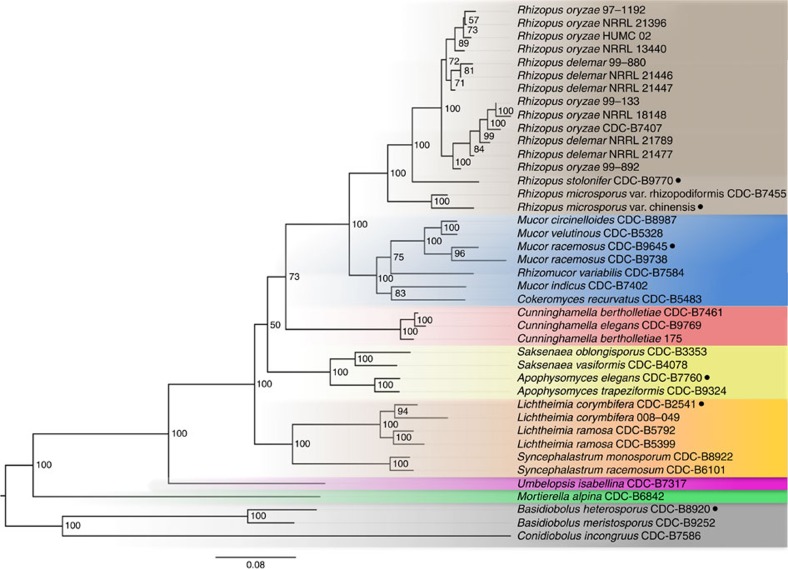
Phylogenetic relationships among 38 Mucorales fungi with three Entomopthorales as outgroups. A phylogenetic tree was constructed using concatenated trimmed multiple alignments of 76 orthologous proteins shared among all 41 fungal strains. Bootstrap values (out of 100) are displayed at tree nodes. Of note, the *R. oryzae* and *R. delemar* sub-clade contains low bootstrap values throughout. All strains are clinical isolates unless otherwise indicated by a black circle to be environmental.

To explore the phylogenetic relationship among the 41 isolates, we identified 76 single-copy core genes present in all 41 genomes and estimated the phylogeny of these organisms using PhyML v3.0 (ref. 27; Fig. 1). As expected, the three Entomopthorales genomes formed a distinct grouping separate from the other 38 genomes. *M. alpina* appears to be in a group by itself near the base of the tree, as does *Umbelopsis isabellina*. The remainder of the isolates, all of which are Mucorales, formed five distinct clades. One such clade was made up completely of *Rhizopus* isolates, while another clade included *Mucor*, *Rhizomucor* and *Cokeromyces* isolates. A third clade contained only *Cunninghamella*. Isolates of *Saksenaea* and *Apophysomyces* clustered together into a fourth clade, and the *Lichtheimia* and *Syncephalastrum* isolates formed the final clade. Overall, our results largely agree with the phylogenetic analysis performed by Hoffmann *et al*.^28^ using four molecular markers.

Unexpectedly, *Mucor racemosus* strain 97-1192 grouped with the *Rhizopus oryzae* and *R. delemar* strains, consistent with the idea that strain 97-1192 was originally misclassified as a *Mucor* and actually belongs to the genus *Rhizopus* as either *oryzae* or *delemar*. From this point forward, we will refer to strain 97-1192 as a *Rhizopus* strain. This strain was included in a more specific phylogenetic analysis as discussed below.

We were also surprised that *R. microsporus* strain CDC-B9738 grouped together with the *Mucor* and *Rhizomucor* isolates and, based on our analysis, is most closely related to *M. racemosus* CDC-B9645 (Fig. 1). We believe that CDC-B9738 was also misclassified and from this point forward we will refer to strain CDC-B9738 as *M. racemosus*.

### Expansion of chitin deacetylases in Mucorales

We used an unbiased approach to identify recurring protein motifs that were present in our predicted proteomes using MEME, a program that finds novel, ungapped motifs (recurring, fixed-length patterns) in protein or nucleotide sequences^29^. Searching the predicted *R. delemar* 99-880 proteome with MEME revealed a number of conserved motifs, which were present throughout dozens of proteins. The sequence logos for 20 most significant motifs are depicted in Supplementary Fig. 1 and listed in Supplementary Data 3. The most significant motif discovered was annotated by our pipeline as a member of the chitin deacetylase (CDA) family of proteins (Supplementary Data 3). A hidden Markov model (HMM) built from a multiple sequence alignment of these proteins (Supplementary Fig. 2a) was searched against 92 widely divergent fungal genomes, and all except *Schizosaccharomyces* or *Enterocytozoon* had at least one peptide that was similar (Supplementary Fig. 2b). Our results are consistent with previous reports showing the expansion of this gene family in a single strain, *R. delemar* strain 99-880 (refs 20, 30). However, this chitin deacetylase family also appears expanded in the other Mucoralean fungi in the present study, although it does appear restricted to the early diverging fungal lineage. The functional consequences of the CDA expansion are unclear.

### Expansion of invasin-encoding genes

**Figure 2:**
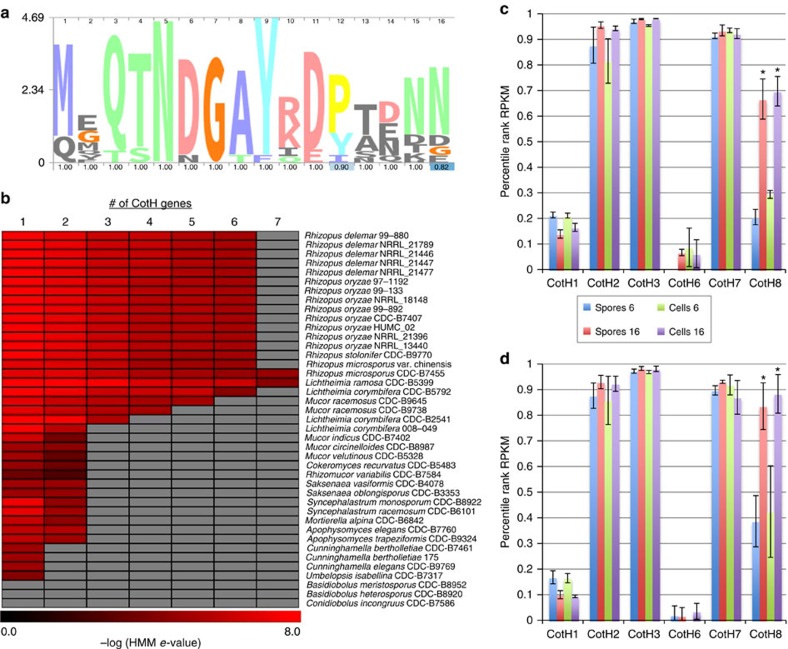
Presence of CotH invasins across Mucorales. (**a**) HMM logo depicting conserved invasin domain. (**b**) Presence of CotH3 domain containing proteins in all 41 Mucorales fungi. Values represent –log of the *e* value from an HMM search of each positive hit (maximum *e* value of 1 × 10^−4^) versus the motif from **a**. Grey colour indicates no gene. (**c**,**d**) Relative gene expression levels (expressed as percentile rank RPKM for all of the genes in the genome) for the six CotH genes of *R. delemar* 99-880 (**c**) and *R. oryzae* 99-892 (**d**) during *in vitro* infection conditions. Blue and red bars indicate spores grown alone in tissue culture media for 6 h (blue) or 16 h (red). Green and purple bars indicate spores grown exposed to airway epithelial cells for 6 h (green) or 16 h (purple). Results are the mean±s.d. *P<0.05 versus 6-h sample in the same condition; *n*=3. Gene of the same name in R. *delemar* 99-880 and *R. oryzae* 99-892 represent 1-to-1 homologues.

We have previously described the identification of three surface proteins encoded by *R. delemar* 99-880, CotH1, CotH2 and CotH3. The latter two function as invasins to promote infection^31^. We used our genomic data set to probe for the presence of CotH-like proteins in all 41 annotated genomes presented here. An HMM was built using the BLASTP hits from each genome against the CotH motif ‘MGQTNDGAYRDPTDNN' (Fig. 2a). This motif is a surface-exposed region against which a therapeutic antibody has been raised^31^. We then identified proteins encoded in each genome that both contained the HMM sequence and had a BLAST *e* value of <1*e*^−20^. The most commonly isolated species from mucormycosis infections, *Rhizopus* spp., each contained 6–7 CotH gene copies. The *Mucor*, *Rhizomucor*, *Cokeromyces* and *Lichtheimia* isolates, which are also commonly isolated as the cause of mucormycosis, contained 3–7 copies depending on the isolates. The *Apophysomyces*, *Cunninghamella*, *Saksenaea*, *Syncephalastrum, Mortierella* and *Umbelopsis* isolates, which are rarely isolated from mucormycosis patients, contained only 1–2 copies. Lastly, the Entomopthorales isolates, which do not cause invasive disease in humans, do not contain any homologues of the CotH-like genes (Fig. 2b). Taken together, these results suggest that, when considering the genome content of Mucoralean fungi, the presence and number of CotH-like genes correlates with clinical prevalence and the ability to cause invasive disease. Future studies will evaluate the relationship between the number of CotH genes in the genomes and the ability to invade and damage host cells.

Using this genomic approach, we have identified three additional CotH paralogues in the *R. oryzae* and *R. delemar* isolates. These paralogs are named CotH6 (IGS-990-880_03186)^20^, CotH7 (IGS-990-880_09445)^20^ and CotH8 (IGS-990-880_11474)^20^ because we previously identified CotH4 and CotH5 (ref. 31). The CotH4 and CotH5 were identified by their relative low identity to the entire CotH3 sequence (20–24% identity at the amino-acid level), but they lack the sequence motif ‘MGQTNDGAYRDPTDNN' used in this study. The messenger RNA (mRNA) expression levels of CotH1-3 and CotH6-8 genes in *R. delemar* 99-880 and *R. oryzae* 99-892 were examined using RNA sequencing (RNA-seq) data from these strains grown in the presence or absence of host airway epithelial cells. While none of the six genes are specifically induced by the presence of host cells, three of the six CotH-like genes (CotH2, CotH3 and CotH7) are among the most highly expressed genes in the genome in all four conditions, and a fourth (CotH8) seems to increase in expression with extended time in tissue culture media. Specifically, during the 6 to16-h assay period, the *R. delemar* CotH8 gene is induced 3.2-fold and 2.3-fold in the absence and presence of host cells, respectively. Similar results were obtained for *R. oryzae* CotH8 gene with induction of 2.1-fold and 2-fold in the same comparisons (Fig. 2c,d). The high expression of these unique CotH genes attests to their importance in Mucorales.

### Population structure of *Rhizopus* strains

**Figure 3:**
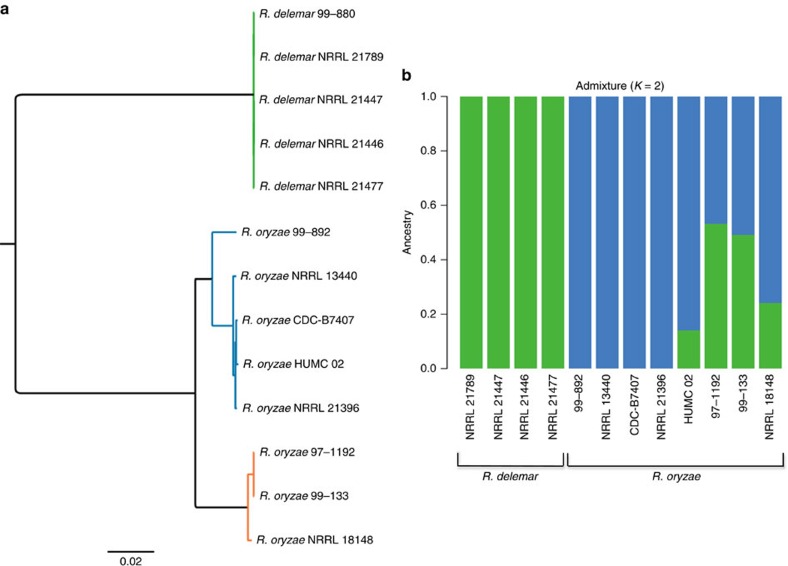
Population structure of *R. oryzae* and *R. delemar* strains. (**a**) SNP-based whole-genome maximum-likelihood phylogeny of all 13 *R. delemar* and *R. oryzae* strains. All nodes have a bootstrap value of ≥97 out of 100. (**b**) Population structure inferred using the program Admixture. Values represent fraction of population ancestry denoted by colours: green (*R. delemar*) and blue (*R. oryzae*).

*R. oryzae* (syn. *Rhizopus arrhizus*) was reclassified into *R. oryzae* and *R. delemar* based on the ability to produce lactic acid and fumaric–malic acids, respectively^32^. The difference in the small organic acid production between these groups lies in the fact that *R. oryzae* strains contain a copy of LdhA, encoding lactate dehydrogenase, while *R. delemar* strains lack a copy of LdhA^32^. However, it has been argued that these differences are insufficient to support separate species and that varieties *R. arrhizus* var. *arrhizus* and var. *delemar* should instead be used^33^,^34^. For the sake of clarity, we use the previous nomenclature proposed by Abe *et al*.^32^ Our phylogeny presented in Fig. 1, based on a limited number of proteins provided poor resolution of the *R. oryzae* and *R. delemar* strains as evidence by the low bootstrap values. To better resolve the population structure, we performed whole-genome phylogeny for all of our *R. delemar* and *R. oryzae* genome sequences, including reclassified strain 97-1192. In total, 2,492,513 single-nucleotide polymorphisms (SNPs) were detected among all 13 isolates, of which 1,620,389 filtered SNPs present in all genomes were retained for tree building. Figure 3a depicts the SNP-based whole-genome phylogeny. Interestingly, the isolates formed three clades instead of two clades as previous phylogenies have suggested^32^,^35^ (Fig. 3a). As expected, one clade contained only *R. oryzae*, while another clade contained only *R. delemar* isolates. The new third clade contained one strain that has been classified as *R. oryzae*, one strain classified as *R. delemar* and strain 97-1192 (formerly *M. racemosus*). Henceforth, we will refer to strains 99-133, NRRL 18148 and 97-1192 as *R. oryzae*. The possibility of two cryptic species within the *R. oryzae* group was previously recognized with phylogenic analysis of ITS, EF-1α and AFLP, but not *act1*; however, the lack of phenotypic differences prevented them from placing species limits within this group^32^. The SNP-based whole-genome phylogeny more clearly defines these clades than phylogenetic analysis with single and combined gene fragments.

We next examined whether the *R. oryzae* (clade 3) isolates are the products of mating between *R. oryzae* and *R. delemar* strains. To address this, we performed admixture analysis on all 13 *oryzae/delemar* strains. The *K*=2 model, which has the lowest cross-validation error, indicates that the clade 3 individuals are admixed ‘mosaics' with some ancestry from each of *R. oryzae* and *R. delemar*, and that *R. oryzae* strain HUMC 02 also has some ancestry from the clade containing the *R. delemar* strains (Fig. 3b). While zygospore formation with *in vitro* mating has been demonstrated between *R. oryzae* and *R. delemar*^35^, it is unclear if zygospores germinate to produce viable progeny or if recombination has actually occurred. The small sample size warrants consideration of the *K*=3 model that only has a slightly higher cross-validation error (Supplementary Fig. 3). This model indicates a third clade, consistent with our whole-genome phylogeny, but indicates that one strain (NRRL 18148) has ancestry from both clades 1 and 3. While both models show evidence of mixed ancestry, more information is needed to fully test these competing hypotheses.

To determine if the strains in each of the three clades vary in their virulence potential, we tested each of the strains for the ability to damage epithelial and endothelial cells during *in vitro* infection of monolayers. All of the strains tested, including representatives from each of the three clades, induced equivalent amounts of damage to both cell types (Supplementary Fig. 4). We also tested representatives from each of the three clades in a neutropenic mouse model of mucormycosis and observed no significant difference in the survival curves (Supplementary Fig. 5). Taken together, these results suggest that there are no clade-associated differences in the ability of these strains to cause disease.

### Identification of *Rhizopus*-specific genes

**Figure 4:**
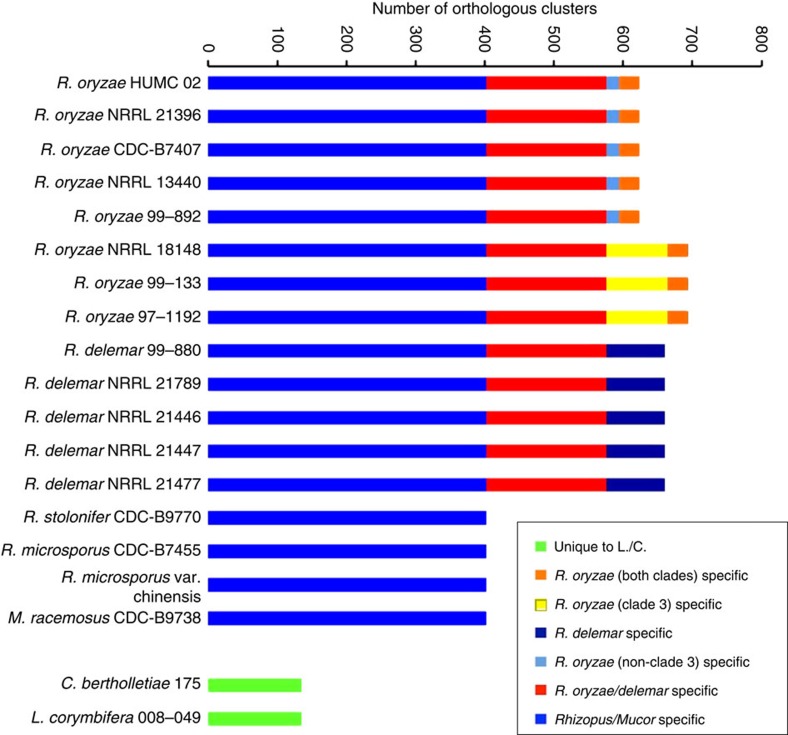
Identification of orthologous clusters. A bar plot of orthologous gene clusters in a subset of 19 Mucorales fungi. Core genes found in all 17 *Rhizopus* and *Mucor* strains are shown in blue, *R. delemar/oryzae*-specific genes are shown in red. Please refer to key for other classifications.

Since *R. oryzae* is most commonly isolated from patients with mucormycosis, we hypothesized that its high clinical prevalence can be explained by differences in genomic content as compared with other Mucorales species. To accomplish this, we built a comparative database and searched for Jaccard orthologous clusters. Our database included the genome annotations from 13*R. oryzae/delemar* strains, two *R. microsporus* strains, one *R. stolonifer* strain, one *M. racemosus*, one *L. corymbifera* strain and one *Cunninghamella bertholletiae* strain. We identified 2,658 ‘core' clusters that are present in all the 19 the genomes. To identify putative virulence genes, we took a conservative approach and searched for clusters that are present only in the 13 *oryzae*/*delemar* strains and absent from each of the other six genomes. Since, we were unable to identify any differences in pathogenic potential *in vitro*, we reasoned that such virulence genes should be present in all the 13*R. oryzae/delemar* strains reported here (Fig. 4). We identified 174 clusters of putative virulence genes that meet our criteria (Supplementary Data 4). We chose to further focus our list of putative virulence genes using two criteria, the presence of secretion signal and high mRNA expression, those genes whose average number of reads per kilobase of exon per million mapped reads (RPKM) ranked in the top 25% of all genes. We reasoned that genes encoding proteins with putative secretion signals might be surface exposed and directly involved in the interaction between the fungus and the host cell. Indeed, in our analyses, CotH3 has a predicted secretion signal and is known to govern pathogenesis^31^. Furthermore, *in vivo* infection studies with *Candida albicans* have suggested a correlation between gene expression and involvement in virulence without consideration for whether a gene's expression is induced by interaction with the host cell^36^,^37^. We acknowledge that other criteria can also be used to identify putative virulence genes; however, with the general scarcity of molecular pathogenesis data for Mucorales, we decided to use these criteria that have been demonstrated for other fungal pathogens.

Since, we have extensive RNA-seq data sets for *R. oryzae* 99-892 and *R. delemar* 99-880 grown in the presence and absence of airway epithelial cells, we focused on these two strains. From 174 clusters, we identified six genes that meet these criteria (Supplementary Data 5). Many of the genes, including all six that have secretion signals, encode hypothetical proteins and serve as excellent candidates for virulence genes that might explain the superior clinical prevalence of *R. oryzae* and *R. delemar*. Further experiments are required to determine the role of these genes in pathogenesis.

### Dual species RNA-seq of *in vitro Rhizopus* infection

To date, there have been no large-scale studies to examine how Mucorales fungi and host cells respond to one another during the course of infection. To this end, we performed RNA-seq on poly(A)-enriched RNA isolated from human airway epithelial cells (A549) that had been infected with *R. delemar* (strain 99-880) or *R. oryzae* (strain 99-892) for 6 or 16 h. The RNA preparations contained a mixture of mRNAs expressed by the *Rhizopus* spp., as well as by the host cells. To ensure that the observed fungal gene expression changes were due to the interaction with the host cell and not simply a response to the medium, we performed RNA-seq on time-matched controls in which each *Rhizopus* isolate was grown in each type of tissue culture medium in the absence of host cells. Similarly, we performed RNA-seq on control, uninfected airway epithelial cells.

We defined differentially expressed genes, as those with a minimum of two-fold change in gene expression (*P*<0.05) between *Rhizopus*-infected host cells compared with either uninfected host cells or cells grown in the corresponding tissue culture medium in the absence of host cells. Each of the *Rhizopus* strains tested mounted a transcriptional response to the airway epithelial cells. *R. delemar* 99-880 displayed 459 and 583 differentially expressed genes at 6 and 16 h post infection, respectively (Supplementary Data 6). R. ory*zae* 99-892 displayed 363 and 90 genes differentially expressed at 6 and 16 h post infection, respectively (Supplementary Data 7). The most highly induced genes in R. delemar 99-880, at after 6 h of infection, are a hypothetical protein containing a motif with transporter functions. This gene (IGS-99-880_01649) displayed significant similarity to human MFSD1 (major facilitator superfamily domain containing 1). These data provide several target genes for functional follow-up experiments.

**Figure 5:**
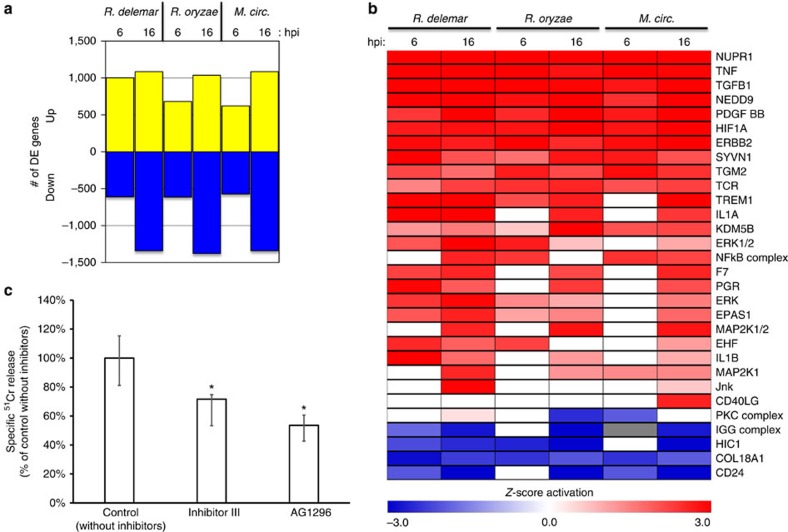
Host response to Mucorales fungi. (**a**) The number of host genes that were induced (yellow) or repressed (blue) by infection with each of the Mucorales fungi at 6 or 16 h after addition of fungal spores. (**b**) Host upstream regulators that are predicted to be modulated by Mucorales infection of airway epithelial cells based on expression of known downstream targets. Red indicates predicted activation (*z* score>2). Blue indicates predicted repression (*z* score<−2). White indicates no predicted regulation. (**c**) The effect of pharmacological inhibition of the PDFG receptor on *R. delemar* 99-880-induced cell damage of endothelial cells. Results are the median±interquartile range of two experiments, each performed in triplicate. *P<0.018 versus control; *n*=6.

For the host analysis, in addition to *R. orzyae* 99-892 and *R. delemar* 99-880, we also examined the response of A549 cells to exposure to *M. circinelloides* NRRL 3631 for 6 and 16 h. Analysis of host cell gene expression revealed a robust response to each of the three Mucorales strains (Supplementary Data 8), each of which induced equivalent amounts of differential host gene expression (Fig. 5a). We have previously shown that considerable insights into disease pathogenesis can be obtained by inferring signal transduction pathways based on gene expression changes^38^. Specifically, we used the Upstream Regulator Analytic from the Ingenuity Pathway Analysis software (Ingenuity systems; http://www.ingenuity.com) to identify signalling proteins whose downstream pathways were potentially activated or repressed during the course of infection with each of the *Rhizopus* strains (Supplementary Data 9). Despite some minor difference, each of the Mucorales fungi elicited similar host signalling profiles (Fig. 5b). Our analysis predicted the activation of several signalling pathways that are well established as part of the host response to fungal pathogens including: tumour necrosis factor, interleukin-1 alpha, interleukin-1 beta, nuclear factor kappa B and mitogen-activated protein kinase 1(refs 39, 40, 41, 42). Our analysis also predicted the activation of pathways that were recently shown to be part of the host response to *C. albicans* infection including: ERBB2 (erbb2 tyrosine kinase 2), NEDD9 (neural precursor cell expressed, developmentally down-regulated 9), PDGF BB (BB homodimer), SYVN1 (synovial apoptosis inhibitor 1) and NUPR1 (nuclear protein, transcriptional regulator 1)^38^,^43^. Given that Ascomycetes (including *C. albicans*) and Mucorales (including *Rhizopus* and *Mucor* spp.) are thought to have diverged from a common ancestor over 800 Myr ago^44^,^45^,^46^, these results suggest the existence of a core host response to fungal pathogens.

We were particularly interested in the predicted activation of the PDGF BB pathway in infection because of the angioinvasive nature of mucormycosis. PDGFs are serum proteins that stimulate cellular migration and have well-established roles in angiogenesis and human diseases, such as cancer and atherosclerosis^47^,^48^,^49^,^50^. The PDGFs are encoded by four different genes—PDGFA, PDGFB, PDGFC and PDGFD—and function as secreted homodimeric or heterodimeric proteins that bind to, and induce the tyrosine phosphorylation of, the PDGFR subunits^47^. The α and β PDGFR subunits can combine to form homodimeric or heterodimeric receptors^51^. We observed a significant overlap between the set of genes that are induced in response to infection with different Mucorales strains and the set of genes that are known to be regulated by the interaction of the PDGF BB homodimer with its receptor. PDGF BB homodimers can bind to and activate signalling via the receptor α/α homodimers, β/β homodimers or α/β heterodimers^51^. These results are consistent with PDGFR phosphorylation and subsequent activation of cellular signalling through either the PDGFRA or PDGFRB subunits. To test whether signalling through PDGFR subunits governs the interaction between Mucorales fungi and host cells, we analysed the effects of two different small molecule PDGFR tyrosine kinase inhibitors on *R. delemar*-induced cell damage of endothelial cells. Both AG1296 (ref. 52) and PDGFR tyrosine kinase inhibitor III (CAS 205254-94-0)^53^ significantly reduced cell damage of endothelial cells induced by infection with *R. delemar* (Fig. 5c). These results suggest that Mucorales strains use PDGF signalling as a mechanism to damage barrier host cells and that *Rhizopus* likely induces expression of angiogenesis pathways to aid in its hematogenous dissemination.

Fungal cell adherence and endocytosis both contribute to the ability of Mucorales strains to cause host cell damage. PDGFR has been shown to promote endocytosis of both *C. albicans* and *Chlamydia trachomatis*^54^. While we have not specifically tested adherence or invasion in this study, further experiments are under way to elucidate the molecular mechanism underlying the reduction of damage conferred by the PDGFRB inhibitors. Although we observed significant reduction in the induction of damage upon chemical inhibition of PDGFRB, host cell damage was not completely abrogated. This might be explained by the fact that *R. delemar* is known to interact with host cell receptor GRP78 to induce its own endocytosis^31^,^55^. Another intriguing possibility is that *R. delemar* can also use ErbB2 as a receptor to induce endocytosis. On the basis of our transcriptional analysis, the ErbB2 signalling pathway is predicted to be activated by Mucorales infection. During *C. albicans* infection, ErbB2 signalling is predicted to be activated and inhibition of signalling reduces fungal endocytosis by the host cells^38^,^43^.

Our combination of comparative genomic and RNA-seq analyses of both fungal and host transcription during *in vitro* infections provides a framework for understanding the molecular pathogenesis of this increasingly important class of fungi. Specifically, we provide evolutionary insight into how pathogenesis might have evolved among this class of fungi and have identified a core host pathway that can be pharmacologically inhibited to reduce damage caused by phylogenetically distinct fungi.

## Methods

### Strains and genome sequencing

In this study, we have sequenced, assembled and annotated the genomes of 30 fungal isolates (Table 1; Supplementary Data 1); we annotated two additional isolates sequenced previously by others (*R. delemar* 99-880 and *R. microsporus* var *chinensis*); and we included in comparative analyses nine isolates previously published by us (Supplementary Data 1). Taxa included: *A. elegans* CDC-B7760, *Apophysomyces trapeziformis* CDC-B9324, *B. heterosporus* CDC-B8920, *B. meristosporus* CDC-B9252, *Cokeromyces recurvatus* CDC-B5483, *C. incongruus* CDC-B7586, *C. bertholletiae* CDC-B7461, *C. bertholletiae* 175, *Cunninghamella elegans* CDC-B9769, *L. corymbifera* 008-049 (ref. 16), *L. corymbifera* CDC-B2541 (ref. 16), *L. ramosa* CDC-B5792 (ref. 16), *L. ramosa* CDC-B5399 (ref. 16), *M. alpina* CDC-B6842 (ref. 26), *M. circinelloides* CDC-B8987 (ref. 22), *M. indicus* CDC-B7402 (ref. 22), *M. racemosus* CDC-B9645 (ref. 22), *Mucor velutinous* CDC-B5328 (ref. 22), *Rhizomucor variabilis* CDC-B7584, *R. delemar* 99-880 (ref. 20), *R. delemar* NRRL 21446, *R. delemar* NRRL 21447, *R. delemar* NRRL 21477, *R. delemar* NRRL 21789, *R. microsporus* CDC-B7455, *M. racemosus* CDC-B9738, *R. microsporus* var *chinensis* (ref. 25)*, R. oryzae* 99-892, *R. oryzae* CDC-B7407, *R. oryzae* HUMC 02, *R. oryzae* NRRL 13440, *R. oryzae* NRRL 21396, *R. stolonifer* CDC-B9770, *Rhizopus* species 97-1192, *Rhizopus* species 99-133, *Rhizopus* species NRRL 18148, *Saksenaea oblongisporus* CDC-B3353, *Saksenaea vasiformis* CDC-B4078, *Syncephalastrum monosporum* B8922, *Syncephalastrum racemosum* B6101 and *U. isabellina* B7317 (ref. 16).

DNA was extracted from fungi grown on Sabouraud's dextrose agar using the GeneRite kit (Carlsbarg, CA) or the OmniPrep kit (GBiosciences). The genome sequence of each isolate was generated at the Institute for Genome Sciences (IGS) Genomics Resource Center (Baltimore, MD; http://www.igs.umaryland.edu) using a combination of paired-end libraries (average insert size of 459 bp) and mate-pair (3 kb) libraries on the Illumina HiSeq2000. Draft genomes were assembled using the MaSuRCA v.1.9.2 genome assembler^56^.

### Structural and functional genome annotation

Prediction of gene structures and assigning putative functions were performed at the IGS Informatics Resource Center (Baltimore, MD). Five isolates were grown in the presence of epithelial cell line (A549 adenocarcinomic human alveolar basal cells), human umbilical vein endothelial cells or in mammalian tissue culture media alone, and for each the following number of RNA-seq reads were generated: *L. corymbifera* 008-049, 439 million (previously described in Chibucos, *et al*.^16^); *C. bertholletiae* 175, 409 million; *R.* oryzae (clade 3) 97-1192 (formerly *Mucor*), 477 million; *R. delemar* 99-880, 412 million; and *R. oryzae* 99-892, 459 million. RNA-seq reads for a given strain were pooled and RNA-seq assemblies, both *de novo* and genome-guided against genomic scaffolds, were generated with Trinity^57^. Both types of assemblies were mapped to their respective genomes (for example, *R. delemar* 99-892 RNA-seq reads against *R. delemar* 99-892 genome) using PASA^58^, and *de novo* assemblies were mapped to other closely related genomes (for example, *R. delemar* 99-880 against the other *R. delemar* genomes) with Genomic Mapping and Alignment Program^59^. Genomic repeat regions were annotated and masked using RepeatMasker to detect repeats from a database of known repeats and RepeatModeler along with RepeatMasker to discover repeats *de novo*. Protein-coding genes were predicted *ab initio* with CEGMA, GeneMark-ES, Augustus, SNAP, GlimmerHMM and GeneID. Augustus, SNAP and GlimmerHMM used CEGMA predictions for parameter training, and GeneID used a parameter file generated by CEGMA. Raw RNA-seq reads were used to augment Augustus training for *C. bertholletiae* 175, *L. corymbifera* 008-049 (previously described in Chibucos, *et al*.^16^), *Rhizopus* species 97-1192 (formerly *Mucor*), *R. delemar* 99-880 and *R. oryzae* 99-892. Spliced alignments of SwissProt proteins against each genome were generated with AAT using cutoffs of 80% similarity and 1,500-bp max intron length. To generate a consensus gene model set, all intrinsic and extrinsic predictions were combined with Evidence Modeller using the following evidence weights: CEGMA 4, Augustus 4, GeneMark-ES 2, GlimmerHMM 2, SNAP 2, GeneID 2 and AAT alignments 2. Assembled RNA-seq transcript alignments were weighted 10 for alignment to self (for example, *R. oryzae* 99-892 transcripts aligned with PASA to *R. oryzae* 99-892 genome), but weighted 1 when aligned to other (for example, *R. oryzae* 99-892 transcripts aligned with Genomic Mapping and Alignment Program to *R. oryzae* 99-133). Non-coding RNAs were predicted with tRNAScan-SE and RNAmmer.

A Jbrowse^60^ instance displaying gene structures and expression data (RNA-seq read coverage) was set up to enable comparison of the *R. delemar* 99-880 gene structures generated in this study with those of the original annotation set^20^. To generate functional assignments, including Gene Ontology terms and Enzyme Commission numbers, predicted proteins were compared with UniProt with BLAST (*e* value of 1*e*^−20^) and against PFAMs/TIGRFAMs with HMM searches (using cutoff values intrinsic to each model). To identify putatively secreted proteins by the presence and location of signal peptide cleavage sites, SignalP v.4.1 (ref. 61) was run on predicted proteomes.

### Phylogenetic analysis

Each of 41 predicted proteomes was searched against all the others using the NCBI blastall 2.2.21 program blastp with an *e* value of 1*e*^−40^ and BLOSUM62 substitution matrix. BLAST results stored as XML output (−m 7) were filtered with the blast_parser.pl script of the InParnoid v4.1 program^62^ using a bitscore cutoff of 50, and orthologues were detected between every strain pair using InParanoid (bitscore=50; no outgroup; no bootstrapping; other parameters default). Orthologues shared among 41 strains were determined by clustering InParanoid outputs with QuickParanoid (http://pl.postech.ac.kr/QuickParanoid/), resulting in 373 protein families. Following orthologue family detection, any families where all *Rhizopus* proteins were not 99 or 100% identical in length were removed, to give 76 protein families in total. Orthologous proteins were aligned using Clustal Omega to produce a gapped alignment for each orthologue group. A conserved block alignment was generated for each alignment by removing poorly aligned positions and divergent regions with Gblocks v0.91b (ref. 63), resulting in 109,895 positions (45.8%) remaining of the intial 240,187 positions. Trimmed individual alignments were concatenated to generate a single larger alignment. Phylogenetic analysis was performed with PhyML v3.0 (ref. 64) with the following parameters: bootstrap replicates=100; compute approximate likelihood ratio test=no; model name=LG; gamma distribution parameter=estimated; 'middle' of each rate class=mean; amino-acid equilibrium frequencies=model; optimise tree topology=yes; tree topology search=NNIs; starting tree=BioNJ; add random input tree=no; optimise branch lengths=yes; and optimise substitution model parameters=yes. The resulting tree was visualized with FigTree v1.4.2(http://tree.bio.ed.ac.uk/software/figtree/).

For the whole-genome phylogeny of the 13 *Rhizopus* strains, SNPs were detected relative to the genome assembly of 99-880 using the *In Silico* Genotyper v.0.16.10_3 (ref. 65) as previously described^66^. The 1,620,389 filtered SNP sites that were identified in all genomes analysed were concatenated and used for phylogenetic analysis. A maximum-likelihood phylogeny with 100 bootstrap replicates was generated using RAxML v.7.2.8 (ref. 67) and visualized using FigTree v.1.4.2.

### Protein queries

The protein motif ‘MGQTNDGAYRDPTDNN', an antigenic and surface-exposed peptide from *R. delemar* 99-880 CotH3 (ref. 31), was searched initially with NCBI BLASTP using default settings against the predicted proteomes of 41 strains. Matching proteins were aligned with Muscle v3.7 and outputs were manually trimmed with AliView to include only the domain. In total 144 aligned sequences with similarity to ‘MGQTNDGAYRDPTDNN' were used to build a HMM with HMMER 3.0. The model was searched against all proteomes, and resulting hits above the HMM's cutoff threshold were used to construct a heat map depicting presence of proteins containing the domain across taxa. HMM logos were generated with Skylign^68^ with stack height representing information content above background frequency and displayed using consensus colours.

### Admixture analysis

Whole-genome sequencing reads for 12*R. oryzae*/*delemar* samples were aligned to the *R. delemar* 99-880 genomic reference using the Burrows–Wheeler aligner (v0.7.12) alignment tool. These alignment BAM files were utilized as input for GATK (v3.1.1), which included filtering of potential PCR duplicated reads, realignment around indels and fixing mates, to identify genomic variation in each sample when compared with the 99-880 genomics reference. The SNP calling was performed jointly across all 12 samples within a single execution of the ‘Unified Genotyper' variant caller provided by GATK, resulting in the computation of genotypes for 2,392,625 SNP positions across the *R. delemar* 99-880 reference genome. The multi-sample VCF file from GATK was then transformed into the PLINK binary format files using the PLINK (v1.90) tool. PLINK was then used to remove any duplicates SNP positions and perform linkage disequilibrium pruning on the 12 samples to produce a reduced set of unlinked SNPs using the parameter ‘indep-pairwise 50 10 0.1'. This reduced set was used as input for further admixture analysis. The ADMIXTURE (v1.23) tool was used to estimate the proportions of admixture in each of the 12 samples using default settings and varying the number of ancestral populations from 1 to 5 (that is, *k*=1, 2, 3, 4 and 5 ancestral populations). The cross-validation errors were reviewed to determine the two best values for the number of ancestral populations, namely *k*=2 and *k*=3. The estimated admixture proportions based on *k*=2 and *k*=3 were then illustrated through stacked bar plots and compared with the phylogenetic tree constructed for the same 12 samples.

### RNA-seq and gene expression analysis

All RNA-seq libraries (non-strand-specific, paired end) were prepared with the TruSeq RNA Sample Prep kit (Illumina). The total RNA samples were subject to poly (A) enrichment as part of the TruSeq protocol. A sequence of 100 nt was determined from both ends of each complementary DNA fragment using the HiSeq platform (Illumina) per the manufacturer's protocol. Sequencing reads were annotated and aligned to the human genome reference (Ensembl GRCh38), as well as the appropriate *Rhizopus* genome using TopHat2(ref. 69). The alignment files from TopHat2 were used to generate read counts for each gene and a statistical analysis of differential gene expression was performed using the EdgeR package from Bioconductor^70^. A gene was considered differentially expressed if the *P* value for differential expression was <0.05 and the absolute log(base 2)-fold change was ≥1.

### Jaccard clustering

The predicted proteins of 19 Mucorales strains were clustered using a two-phase protein clustering algorithm^71^, in which the first phase defines within-genome putative paralogs (‘Jaccard clusters') and the second employs a transitive reciprocal-best-hit analysis to cluster the Jaccard clusters (rather than individual proteins) across two or more genomes. The algorithm relies on an all-vs-all BLASTP search of the 19 genomes' predicted proteins, and the default parameters were used throughout. The resulting clusters and their genomic context were then examined with Sybil^72^, a web-based software package whose primary aim is to facilitate the analysis and visualization of comparative genome data, with a particular emphasis on protein and gene cluster data.

### *In vitro* infections and inhibitor studies

To study the host response to Mucorales, we incubated the alveolar epithelial A549 cells (ATCC; CCL-185) with *R. oryzae* 99-892, *R. delemar* 99-880 or *M. circinelloides*. A549 cells were grown in 10 × 15-mm tissue culture dish in F-12k medium with L-glutamine (ATCC) supplemented with 10% bovine serum albumin until confluent. The tissue culture medium was F-12 K supplemented with L-glutamine (ATCC), and 2.5 μg ml^−1^ of surfactant, at which time 1 × 0^7^ of each Mucorales were added to each dish for a multiplicity of infection of ∼1.5 cells for every one human cell. After 6 h, or 16 h of infection, the cells were scraped from the plate, and the human and fungal RNA were isolated using the Qiagen RNeasy plant kit according to the manufacturer's instructions. As a negative controls, 1 × 10^7^ Mucorales were added to the tissue culture plates containing medium alone without host cells and processed in parallel. Another control included RNA extracted from A549 cells incubated in parallel without any added Mucorales.

Endothelial cells were collected from umbilical vein endothelial cells by the method of Jaffe *et al*.^73^ and propagated as we described before^31^. Primary human endothelial cell collection was approved by the IRB of the Los Angeles Biomedical Research Institute at Harbor-UCLA Medical Center. Because umbilical cords are collected without donor identifiers, the IRB considers them medical waste not subject to informed consent. *R. delemar*-induced endothelial cell damage was quantified by using a chromium (^51^Cr) release assay^74^. In brief, endothelial cells grown in 96-well tissue culture plates containing detachable wells were incubated with 1 μCi per well of Na_2_^51^CrO_4_ (ICN, Irvine, CA) in M-199 medium for 16 h. On the day of the experiment, the unincorporated ^51^Cr was aspirated, and the wells were washed twice with prewarmed HBSS. PDGFR inhibitors dissolved in 0.2% DMSO in 150 μl RPMI 1640 medium with L-glutamine (Irvine Scientific) were added to endothelial cells for 1 h (final concentration of each inhibitor was 10 μM). Next, the wells were aspirated and a fresh 150 μl of RPMI 1640 containing of the inhibitor and fungal germlings (1.0 × 10^6^ germinated for 1 h) were added and the plate incubated for 4 h at 37 °C in a 5% CO_2_ incubator. Spontaneous ^51^Cr release was determined by incubating endothelial cells in RPMI 1640 containing 0.2% DMSO without the inhibitors or *R. oryzae*. At the end of incubation period, 50% of the medium was aspirated from each well and transferred to glass tubes, and the cells were manually detached and placed into another set of tubes. The amount of ^51^Cr in the aspirate and the detached well was determined by gamma counting. The total amount of ^51^Cr incorporated by endothelial cells in each well equalled the sum of radioactive counts per minute of the aspirated medium plus the radioactive counts of the corresponding detached wells. After the data were corrected for variations in the amount of tracer incorporated in each well, the percentage of specific endothelial cell release of ^51^Cr was calculated by the following formula: ((experimental release × 2)−(spontaneous release × 2))/(total incorporation−(spontaneous release × 2)). Each experimental condition was tested at least in triplicate and the experiment repeated at least once.

### *In vivo* infection

All procedures involving mice were approved by the IACUC of the Los Angeles Biomedical Research Institute at Harbor-UCLA Medical Center according to the NIH guidelines for animal housing and care. The virulence of *R. delemar, R. oryzae* or *R. oryzae* (clade 3) was compared in the neutropenic mouse model of intratracheal infection.^75^ Male ICR mice (6–8-week old and weighing 23–25 g from Taconic Farms, Germantown, NY) were used in this study. Neutropenia was induced by cyclophosphamide (200 mg kg^−1^, intraperitoneal) and cortisone acetate (500 mg kg^−1^, subcutaneous) on day −2 and +3 relative to infection. This treatment regimen results in ∼10 days of leukopenia (day −2 to day +7) with total white blood cell count dropping from ∼130,000 cm^3^ to almost no detectable leukocytes as determined by Unopette System (Becton-Dickinson and Co.). Mice were intratracheally infected with 2.5 × 10^5^ spores of either Mucorales after sedation with ketamine and xylazine^75^. To protect against bacterial infection due to neutropenia, mice were treated with ceftazidime (Western Medical Supply) at 5 mg per mouse given subcutaneously from day 0 until day +7 relative to infection.

### Statistical analysis

Differences in fungi–endothelial cell interactions were compared by the non-parametric Wilcoxon rank-sum test. Comparisons with *P* values of <0.05 were considered significant.

### Data availability

All of the raw sequencing data from this study have been submitted to the NCBI SRA database (http://www.ncbi.nlm.nih.gov). The DNA sequencing data have been deposited under accession codes SRP029722, SRP029720, SRP030764, SRP030765, SRP030703, SRP030763, SRP029751, SRP030704, SRP029724, SRP030711, SRP030715, SRP029744, SRP029718, SRP029712, SRP030712, SRP030762, SRP029709, SRP030713, SRP029747, SRP029758, SRP029597, SRP029749, SRP029754, SRP030769, SRP029746, SRP030760, SRP029750, SRP029759, SRP029879, SRP029755, SRP030719, SRP030710, SRP029741, SRP030708, SRP029743, SRP030717, SRP029717, SRP030716, SRP030709. The RNA sequencing data have been deposited under accession code SRP077731. Refer to Supplementary Data 1 for accession codes for genome sequences. All other supporting data from this study are available within the article and its Supplementary Information files, or from the corresponding authors upon request.

## Additional information

**How to cite this article:** Chibucos, M. C. *et al*. An integrated genomic and transcriptomic survey of mucormycosis-causing fungi. *Nat. Commun.* 7:12218 doi: 10.1038/ncomms12218 (2016).

## Supplementary Material

Supplementary Figures Supplementary figures 1-5

Supplementary Data 1 Isolate Information.

Supplementary Data 2 Sequencing, assembly, and gene summary statistics.

Supplementary Data 3 Top 20 MEME motifs identified.

Supplementary Data 4 Genes only present in *R. delemar* and *R. oryzae* as described in Figure 4.

Supplementary Data 5 Most highly expressed genes in the *R. delemar* (99-880) genome

Supplementary Data 6 List of differentially expressed genes in *R. delemar* 99-880.

Supplementary Data 7 List of differentially expressed genes in *R. oryzae* 99-892.

Supplementary Data 8 Differentially expressed host genes.

Supplementary Data 9 Upstream regulator analysis of host genes' expression.

## Figures and Tables

**Table 1:** List of isolates sequenced in this study.

Genus	Species	No. of Isolates
*Rhizopus*	*R. oryzae*	8
	*R. delemar*	4
	*R. microsporus*	1
	*R. stolonifer*	1
		
*Mucor*	*M. racemosus*	1
		
*Cunninghamella*	*C. bertholletiae*	2
	*C. elegans*	1
		
*Apophysomyces*	*A. elegans*	1
	*A. trapeziformis*	1
		
*Syncephalastrum*	*S. monosporum*	1
	*S. racemosum*	1
		
*Rhizomucor*	*R. variabilis*	1
		
*Cokeromyces*	*C. recurvatus*	1
		
*Saksenaea*	*S. oblongisporus*	1
	*S. vasiformis*	1
		
*Umbelopsis*	*U. isabellina*	1
		
*Basidiobolus*	*B. heterosporus*	1
	*B. meristosporus*	1
		
*Conidiobolus*	*C. incongruus*	1
