# Supplementary material for: An integrated genomic and transcriptomic survey of mucormycosis-causing fungi
Source: Nat Commun. 2016 Jul 22;7:12218. doi: 10.1038/ncomms12218 (PMC4961843; doi:10.1038/ncomms12218)
Supplement: Supplementary Figures — 1-5 [file ncomms12218-s1.pdf]

Figure S1

Logos for top 20 motifs identified using MEME

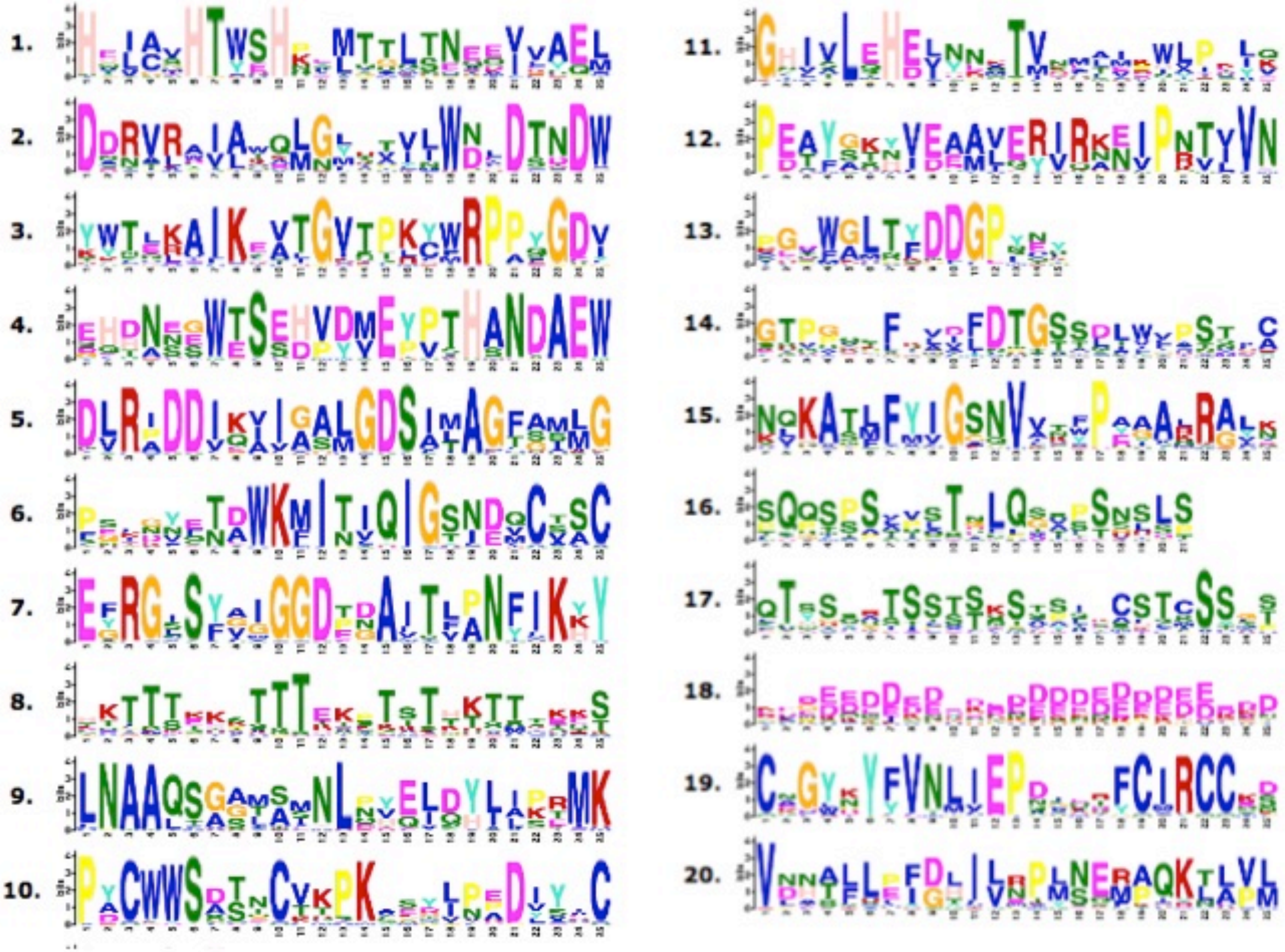

Please refer to Supplementary Table S3 for information about individual motifs.

Supplementary Figure 1. Sequence logos for the 20 most significant motifs identified by our MEME search. Y-axis indicates information content (bits). The numbers refer to motif numbers in Supplementary Data 3 which contains information on individual motifs.

Figure S2

A.

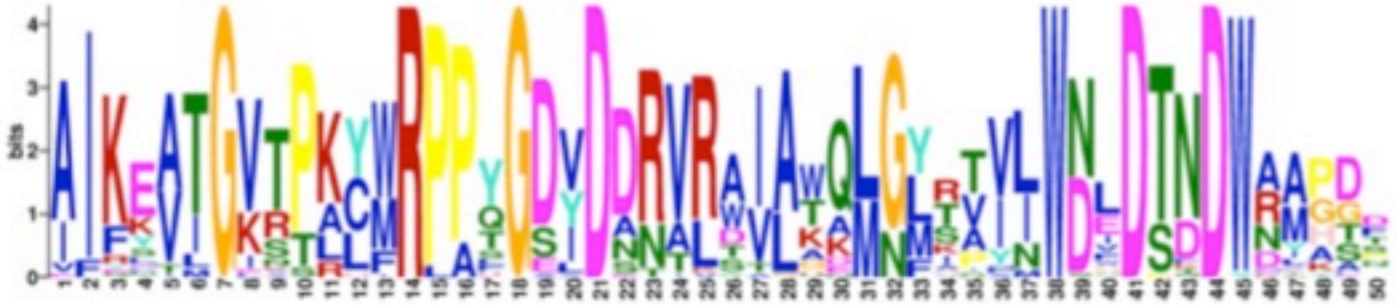
bits

B.

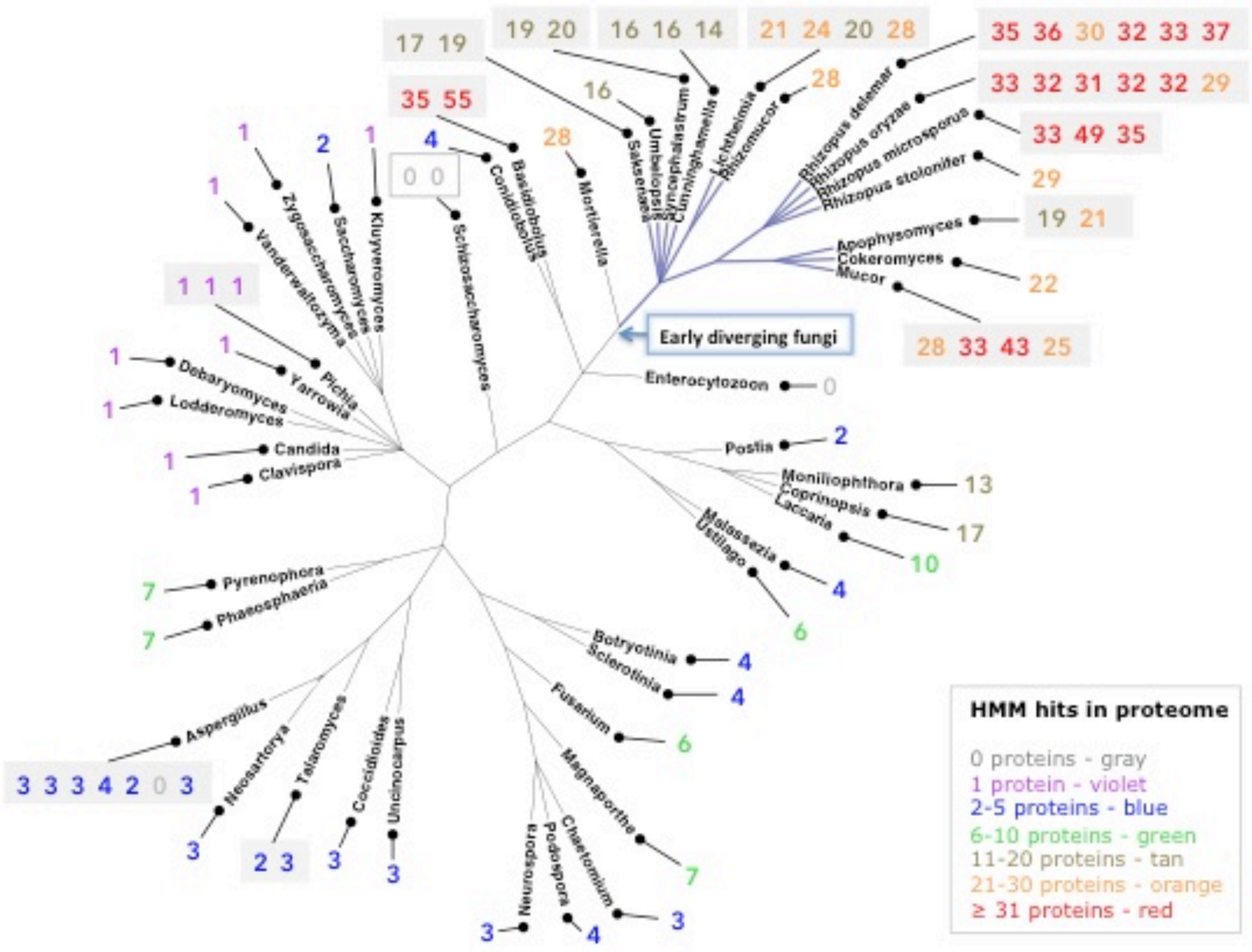
Early diverging fungi
Rhizopus delemar
Rhizopus oryzae
Rhizopus microsporus
Rhizopus stolonifer
Apophysomyces
Cokeromyces
Mucor
Lichtheimia
Rhizomucor
Cunninghamella
Syncephalastrum
Umbelopsis
Saksenaea
Mortierella
Basidiobolus
Conidiobolus
Schizosaccharomyces
Enterocytozoon
Postia
Moniliophthora
Coprinopsis
Laccaria
Malassezia
Ustilago
Botryotinia
Sclerotinia
Fusarium
Magnaporthe
Chaetomium
Podospora
Neurospora
Uncinocarpus
Coccidioides
Talaromyces
Neosartorya
Aspergillus
Pyrenophora
Phaeosphaeria
Kluyveromyces
Saccharomyces
Zygosaccharomyces
Vanderwaltozyma
Pichia
Yarrowia
Debaryomyces
Lodderomyces
Candida
Clavispora
HMM hits in proteome
0 proteins - gray
1 protein - violet
2-5 proteins - blue
6-10 proteins - green
11-20 proteins - tan
21-30 proteins - orange
≥ 31 proteins - red

Supplementary Figure 2. Expansion of chitin deacetylases in Mucorales. A. A putatively secreted chitin deacetylase (IGS-99-880.mRNA.2669.1-Protein) and 37 paralogs were aligned from *Rhizopus delemar* 99-880 and a hidden Markov model (HMM) was built. **B.** A total of 48 divergent fungal genera were searched with the HMM. Number(s) pointing to a given genus represent the number of proteins hitting the model for a species within that genus; multiple numbers for a single genus reflect multiple species sampled.

Figure S3

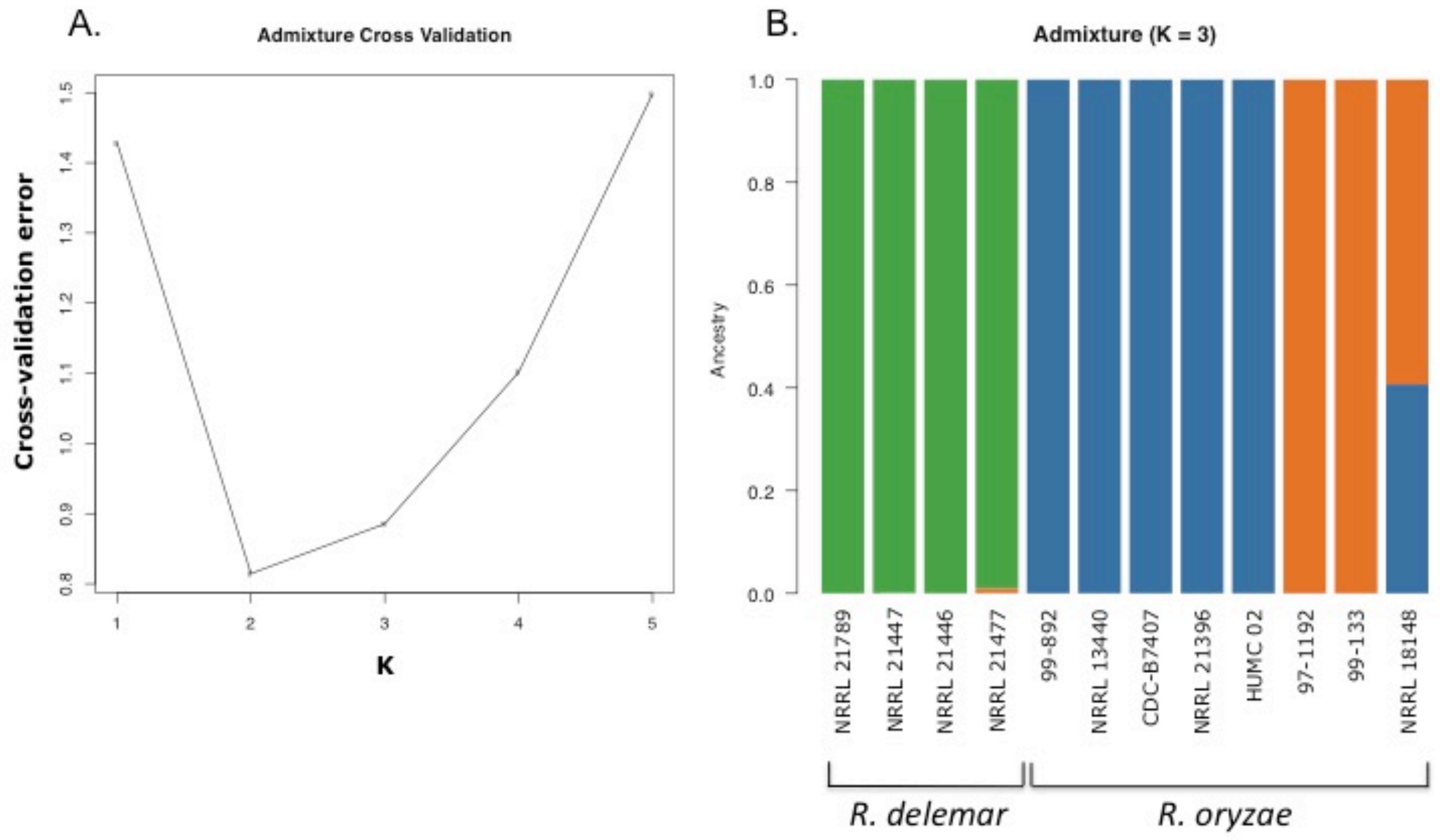
A.
Admixture Cross Validation
Cross-validation error
0.8
0.9
1.0
1.1
1.2
1.3
1.4
1.5
1
2
3
4
5
K
B.
Admixture (K = 3)
Ancestry
0.0
0.2
0.4
0.6
0.8
1.0
NRRL 21789
NRRL 21447
NRRL 21446
NRRL 21477
99-892
NRRL 13440
CDC-B7407
NRRL 21396
HUMC 02
97-1192
99-133
NRRL 18148
R. delemar
R. oryzae

Supplementary Figure 3. Additional Support for Admixture. A. The cross-validation errors as a function of model. The K=2 model had the lowest cross-validation error and was therefore used in the primary analysis **B.** Population structure inferred using the program Admixture using the K=3 model. Values represent fraction of population ancestry denoted by colors: green (*R. delemar*), blue (*R. oryzae*), orange (*R. oryzae* clade 3).

Figure S4

A.

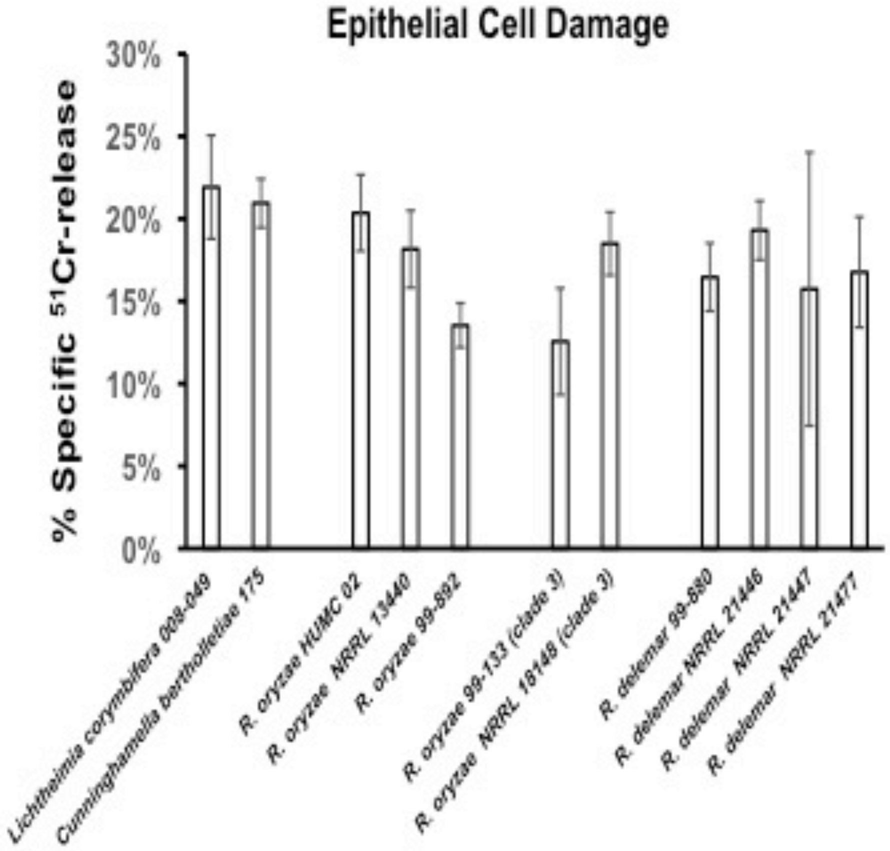
Epithelial Cell Damage
% Specific 51Cr-release
30%
25%
20%
15%
10%
5%
0%
Lichtheimia corymbifera 008-049
Cunninghamella bertholletiae 175
R. oryzae HUMC 02
R. oryzae NRRL 13440
R. oryzae 99-892
R. oryzae 99-133 (clade 3)
R. oryzae NRRL 18148 (clade 3)
R. delemar 99-880
R. delemar NRRL 21446
R. delemar NRRL 21447
R. delemar NRRL 21477

B.

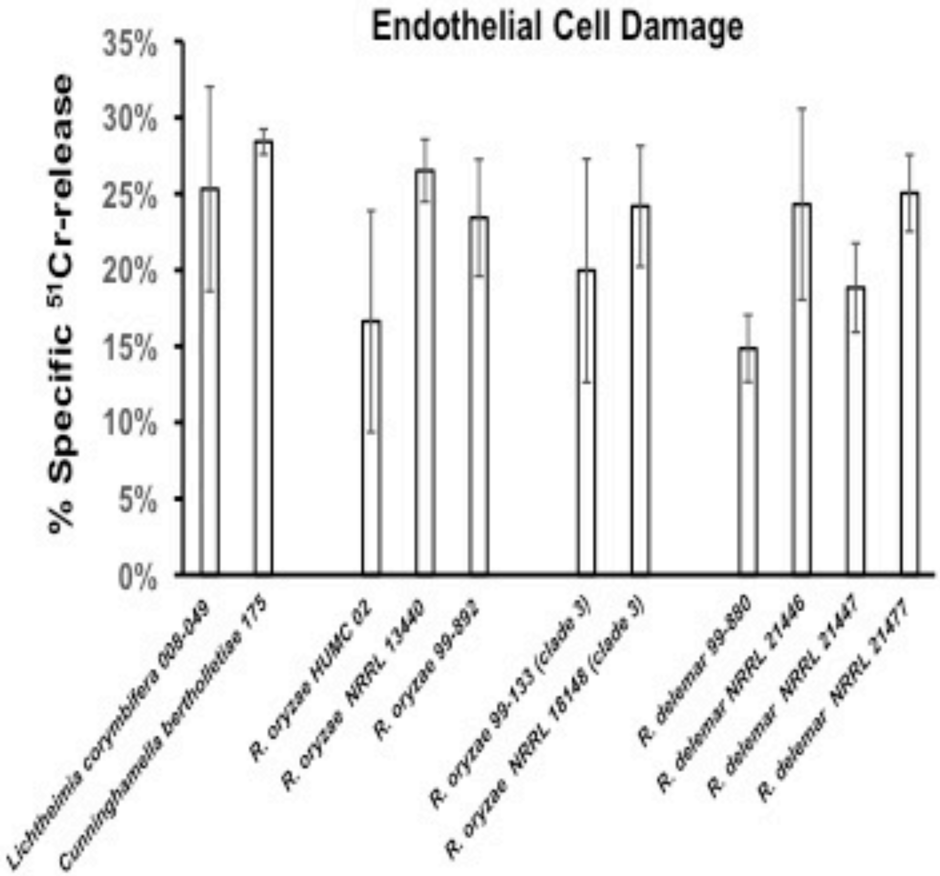
Endothelial Cell Damage
% Specific 51Cr-release
35%
30%
25%
20%
15%
10%
5%
0%
Lichtheimia corymbifera 008-049
Cunninghamella bertholletiae 175
R. oryzae HUMC 02
R. oryzae NRRL 13440
R. oryzae 99-892
R. oryzae 99-133 (clade 3)
R. oryzae NRRL 18148 (clade 3)
R. delemar 99-880
R. delemar NRRL 21446
R. delemar NRRL 21447
R. delemar NRRL 21477

Supplementary Figure 4. Damage of A549 airway epithelial cells (**A**) and human umbilical vein endothelial cells (**B**) caused by representatives of Mucorales investigated in this study. Damage to epithelial cells was carried out for 24 h, while endothelial cell injury was studied at 4 h. No clear differences in damage were observed. Results are the median ± interquartile range; n=6.

Figure S5

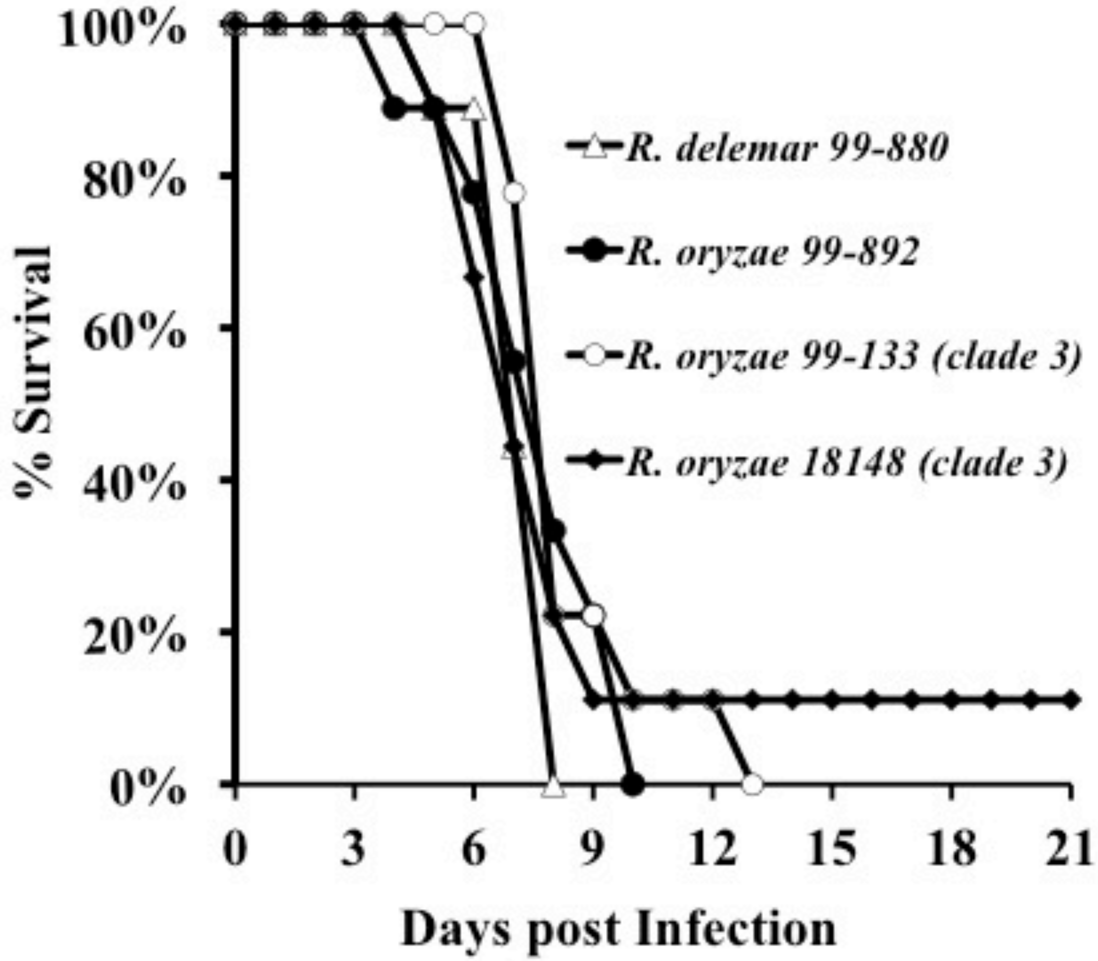

% Survival
100%
80%
60%
40%
20%
0%
0
3
6
9
12
15
18
21
Days post Infection
R. delemar 99-880
R. oryzae 99-892
R. oryzae 99-133 (clade 3)
R. oryzae 18148 (clade 3)

Supplementary Figure 5. ***In vivo*** **infection with** ***R. oryzae*** **and** ***R. delemar*** **isolates.** Survival of mice (n=9 per group) infected intratracheally with 2.5 x 10^5 spores of selected *Rhizopus* strains from each clade described in Figure 3. Confirmed delivered inoculum to the lungs are 2.5 x 10^3 for *R. delemar* 99-880, 4.0 x 10^3 for *R. oryzae* 99-892, 3.3 x 10^3 for *R. oryzae* 99-133 (clade 3), and 2.8 x 10^3 for *R. oryzae* 18148 (clade 3). *P* >0.05 versus all groups by Log Rank test.
