# Supplementary material for: An integrated genomic and transcriptomic survey of mucormycosis-causing fungi
Source: Nat Commun. 2016 Jul 22;7:12218. doi: 10.1038/ncomms12218 (PMC4961843; doi:10.1038/ncomms12218)
Supplement: Supplementary Data 2 — Sequencing, assembly, and gene summary statistics. [file ncomms12218-s3.docx]

**Supplementary Data 2**. Sequencing, assembly, and gene summary statistics. All genome sequence data generated by the Institute for Genome Sciences using Illumina HiSeq technology except: *R. delemar* 99-880, sequenced by the Broad Institute using Sanger ABI; and *R. microsporus* var. chinensis CCTCC M201021, sequenced by the Lab of Brewing Microbiology and Applied Enzymology using Illumina.

| **Taxon** | **Coverage (x)** | **Scaf. no.** | **Scaf. len (Mb)** | **Scaf. N50** | **^§^Scaf. %GC** | **Genes no.** | **Proteins no.** | **rRNA no.** | **tRNA no.** | **CEGMA^** |
| --- | --- | --- | --- | --- | --- | --- | --- | --- | --- | --- |
| ***Rhizopus oryzae*** | | | | | | | | | | |
| NRRL 21396 | 41.53 | 3,003 | 42.89 | 120,603 | 35.2 | 12,243 | 11,967 | 1 | 275 | 98.39, 100 |
| HUMC 02 | 103.09 | 2,315 | 40.4 | 109,804 | 34.6 | 12,264 | 12,144 | 0 | 120 | 95.97, 99.19 |
| CDC-B7407 | 47.77 | 2,553 | 43.43 | 160,365 | 34.9 | 12,418 | 12,151 | 2 | 265 | 98.39, 100 |
| NRRL 13440 | 86.09 | 3,926 | 43.42 | 84,275 | 35.2 | 12,353 | 12,077 | 7 | 269 | 97.98, 99.60 |
| 99-892 | 85.45 | 1,168 | 39.19 | 117,565 | 35.2 | 12,747 | 12,609 | 0 | 138 | 96.77, 98.79 |
| 99-133 | 18.30 | 2,446 | 41.6 | 147,490 | 35.4 | 12,613 | 12,373 | 0 | 240 | 98.39, 100 |
| 97-1192  (formerly *Mucor racemosus*) | 73.11 | 2,946 | 43.00 | 127,402 | 35.3 | 12,275 | 12,001 | 3 | 271 | 97.58, 99.19 |
| ***Rhizopus delemar*** | | | | | | | | | | |
| NRRL 18148 | 22.51 | 11,017 | 47.89 | 289,789 | 35.0 | 14,352 | 14,051 | 0 | 301 | 97.58, 99.19 |
| NRRL 21789 | 64.45 | 2,930 | 42.06 | 87,157 | 35.4 | 11,412 | 11,140 | 0 | 272 | 98.39, 99.19 |
| 99-880 | 14.0 | 81 | 46.09 | 3,104,119 | 35.6 | 13,177 | 12,932 | 6 | 239 | 97.58, 99.19 |
| NRRL 21446 | 75.13 | 1,157 | 39.06 | 156,771 | 35.5 | 12,213 | 12,034 | 1 | 178 | 96.37, 98.39 |
| NRRL 21447 | 80.49 | 1,177 | 38.72 | 143,731 | 35.5 | 12,359 | 12,216 | 1 | 142 | 95.97, 99.60 |
| NRRL 21477 | 80.75 | 1,816 | 40.86 | 206,125 | 34.8 | 12,191 | 12,044 | 0 | 147 | 97.18, 98.79 |
| ***Rhizopus microsporus*** | | | | | | | | | | |
| var. rhizopodiformis CDC-B7455 | 42.75 | 3,730 | 48.84 | 29,877 | 37.2 | 18,545 | 18,269 | 0 | 276 | 96.77, 99.60 |
| var. chinensis CCTCC M201021 | 100.0 | 3,431 | 45.70 | -- | 36.9***** | 17,217 | 16,419 | 4 | 102 | 93.55, 97.58 |
| ***Rhizopus stolonifer*** | | | | | | | | | | |
| CDC-B9770 | 42.23 | 2,812 | 38.24 | 110,603 | 35.5 | 11,108 | 10,844 | 7 | 257 | 94.76, 97.58 |
| **Other Mucorales** | | | | | | | | | | |
| *Apophysomyces elegans* CDC-B7760 | 53.91 | 1,266 | 38.51 | 149,537 | 41.7 | 9,761 | 9,584 | 3 | 174 | 98.79, 99.60 |
| *Apophysomyces trapeziformis* CDC-B9324 | 102.48 | 1,108 | 35.87 | 226,925 | 41.9 | 9,747 | 9,542 | 9 | 196 | 97.58, 98.39 |
| *Basidiobolus heterosporus* CDC-B8920 | 5.21 | 21,535 | 47.63 | 11,409 | 39.7 | 9,331 | 8,992 | 5 | 334 | 81.05, 87.90 |
| *Basidiobolus meristosporus* CDC-B9252 | 22.10 | 18,941 | 103.9 | 18,889 | 41.9 | 13,273 | 13,034 | 13 | 226 | 90.73, 93.55 |
| *Cokeromyces recurvatus* CDC-B5483 | 63.16 | 1,606 | 29.43 | 122,061 | 30.4 | 9,750 | 9,506 | 2 | 242 | 95.16, 95.97 |
| *Conidiobolus incongruus* CDC-B7586 | 75.57 | 5,921 | 95.66 | 43,342 | 22.4 | 17,393 | 16,001 | 13 | 1,379 | 93.55, 95.97 |
| *Cunninghamella bertholetiae* 175 | 84.20 | 655 | 31.40 | 917,193 | 24.8 | 10,177 | 9,992 | 4 | 181 | 98.39, 99.19 |
| *Cunninghamella bertholletia* CDC-B7461 | 82.92 | 628 | 31.16 | 818,170 | 24.7 | 9,689 | 9,542 | 1 | 146 | 98.39, 99.60 |
| *Cunninghamella elegans* B9769 | 115.42 | 1,028 | 31.82 | 663,640 | 24.5 | 8,870 | 8,693 | 1 | 176 | 97.98, 98.79 |
| **℗***Lichtheimia corymbifera* 008-049 | 97.12 | 1,306 | 36.66 | 176,654 | 43.4 | 11,282 | 11,083 | 6 | 193 | 97.98, 98.79 |
| **℗***Lichthiemia corymbifera* CDC-B2541 | 86.17 | 935 | 36.65 | 207,011 | 43.5 | 9,773 | 9,607 | 2 | 164 | 98.39, 98.79 |
| **℗***Lichthiemia corymbifera* CDC-B5792 | 41.57 | 2,782 | 42.52 | 90,825 | 41.1 | 13,746 | 13,483 | 4 | 259 | 95.97, 99.60 |
| **℗***Lichtheimia ramosa* CDC-B5399 | 47.23 | 3,191 | 45.66 | 68,280 | 41.2 | 14,707 | 14,426 | 3 | 278 | 97.58, 99.19 |
| ¶*Mortierella alpina* CDC-B6842 |  | 834 | 39.55 | 424,202 | 50.4 | 11,208 | 10,977 | 0 | 231 | 95.97, 98.39 |
| *§Mucor circinelloides* CDC-B8987 | 100.43 | 1,582 | 36.77 | 91,252 | 39.5 | 10,508 | 10295 | 3 | 210 | 97.58, 99.19 |
| *§Mucor indicus* CDC-B7402 | 55.87 | 1,304 | 39.92 | 270,924 | 35.8 | 11,798 | 11,544 | 0 | 254 | 97.98, 99.60 |
| *§Mucor racemosus* CDC-B9645 | 49.38 | 5,324 | 65.66 | 37,229 | 32.5 | 15,364 | 14,961 | 7 | 396 | 99.19, 100 |
| *Mucor racemosus CDC-9738*  *(formerly Rhizopus microsporus)* | 37.36 | 3,357 | 75.32 | 89,563 | 33.3 | 21,385 | 20,957 | 0 | 428 | 99.19, 100 |
| *§Mucor velutinous* CDC-B5328 | 107.80 | 1,595 | 35.97 | 77,142 | 40.5 | 10,104 | 9,855 | 3 | 246 | 97.58, 98.79 |
| *Rhizomucor variabilis* CDC-B7584 | 59.39 | 681 | 33.44 | 416,810 | 37.8 | 12,032 | 11,811 | 10 | 211 | 97.18, 98.39 |
| *Saksenaea oblongisporus* CDC-B3353 | 72.70 | 1,338 | 40.90 | 197,114 | 41.9 | 9,233 | 9,047 | 3 | 183 | 96.77, 97.98 |
| *Saksenaea vasiformis* CDC-B4078 | 88.08 | 1,772 | 42.57 | 155,085 | 42.7 | 9,852 | 9,656 | 5 | 191 | 97.98, 98.39 |
| *Syncephalastrum monosporum* CDC-B8922 | 75.83 | 821 | 29.61 | 500,002 | 48.3 | 9,046 | 8,910 | 5 | 131 | 98.79, 99.60 |
| *Syncephalastrum racemosum* CDC-B6101 | 73.54 | 700 | 29.59 | 599,572 | 47.2 | 9,162 | 9,019 | 8 | 135 | 98.39, 99.19 |
| *Umbelopsis isabellina* CDC-B7317 | 104.88 | 43 | 21.87 | 1,370,117 | 42.0 | 7,612 | 7,504 | 1 | 107 | 97.58, 99.60 |

§As determined by RepeatMasker on scaffolds

^Percentage of complete models detected, followed by percentage of both complete and partial models detected.

*GC calculated on contigs

**℗**Previously published: Chibucos, M. C. *et al.* The genome sequence of four isolates from the family Lichtheimiaceae. *Pathog Dis* **73**, doi:10.1093/femspd/ftv024 (2015).

¶Previously published: Etienne, K. A. *et al.* Draft Genome Sequence of Mortierella alpina Isolate CDC-B6842. *Genome announcements* **2**, doi:10.1128/genomeA.01180-13 (2014).

*§* Previously published: Shelburne, S. A. *et al.* Implementation of a Pan-Genomic Approach to Investigate Holobiont-Infecting Microbe Interaction: A Case Report of a Leukemic Patient with Invasive Mucormycosis. *PLoS One* **10**, e0139851, doi:10.1371/journal.pone.0139851 (2015).
